# A Survey of Marine Natural Compounds and Their Derivatives with Anti-Cancer Activity Reported in 2010

**DOI:** 10.3390/molecules16075629

**Published:** 2011-06-30

**Authors:** Marc Schumacher, Mareike Kelkel, Mario Dicato, Marc Diederich

**Affiliations:** Laboratoire de Biologie Moléculaire et Cellulaire du Cancer, Fondation de Recherche Cancer et Sang, Hôpital Kirchberg, 9, rue Steichen L-2540, Luxembourg; Email: marc.schumacher@lbmcc.lu (M.S.)

**Keywords:** marine anticancer compounds, natural analogues, synthetic derivatives, cancer

## Abstract

Although considerable progress in oncology therapeutics has been achieved in the last century, cancer remains one of major death causes in the World and for this reason, the development of novel cancer drugs remains a pressing need. Natural marine compounds represent an interesting source of novel leads with potent chemotherapeutic or chemo-preventive activities. In the last decades, structure-activity-relationship studies have led to the development of naturally-derived or semi-synthetic analogues with improved bioactivity, a simplified synthetic target or less toxicity. We aim here to review a selection of natural compounds with reported anticancer activity isolated of marine sources and their associated analogues published in 2010.

## 1. Introduction

Despite the continuous and important advances in biomedical research, the World Health Organization predicts that there will be more then 11 million cancer-related deaths per annum by 2030 [1]. Recent research highlights the isolation of promising compounds with effective anticancer activities from natural sources. An example of these compounds is trabectedin (PharmaMar’s Yondelis^®^) [2], which represents the first anticancer drug isolated from a marine source. Almost 50 percent of the antitumor agents approved over the last 50 years have consisted of compounds either derived from natural sources or (hemi-) synthetic analogues of these products [3]. Natural compounds remain a rich source of promising chemotherapeutic or chemo-preventive agents [4,5,6,7,8].

The sea covers over seventy percent of the Earth’s surface, and ecosystems such as coral reefs contain high levels of biodiversity compared to rainforests. The sea contains many untapped sources of drugs with promising activities due to the extensive variety of marine habitats (influenced by factors such as UV-exposure, the presence of sunlight, and salinity levels) [9]. Over 2700 scientists from over 80 nations, who assessed the diversity, distribution and abundance of marine life, conducted a marine census. The census resulted in the discovery of over 6000 potentially new species [10,11,12,13]. As a consequence of this research effort, it is clear that the marine environment represents a largely unexploited reservoir of unknown natural compounds, which need to be evaluated for potential medicinal applications.

Natural derivatives of potent bioactive compounds from marine organisms can be bio-synthesized. It is well known that in some cases, like that of the plant-derived polyphenol curcumin, the synthetic analog exerts a higher activity compared to the parent compound [14,15]. In addition to natural analogs, chemical modification is an extensive and exceptionally powerful tool for the development of novel drug candidates [16]. Structure-activity-relationship studies of marine compounds can lead to the design of analogs that have greater activity together with a simplified synthetic approach, as reported with bryostatin 1, a compound produced by the marine bryozoan *Bugula neritina*, which has been studied for several years by the Wender research group [17,18].

Many reviews on natural compounds from marine environments have been published [6,19,20,21,22,23,24,25]. Here, however, we will focus only on selected marine anti-cancer agents and analogues either discovered or synthesized in 2010 or whose biological activity was discussed in that year.

## 2. Marine Natural Compounds and Their Derivatives Published in 2010

### 2.1. Cryptosphaerolide *(**1**)*

**Figure 1:**
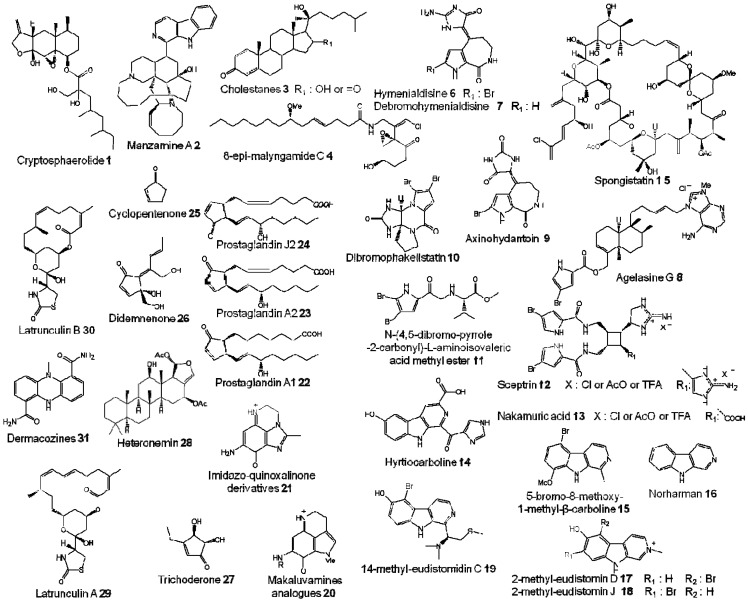
Chemical structures of marine compounds **1–31**.

**Figure 2:**
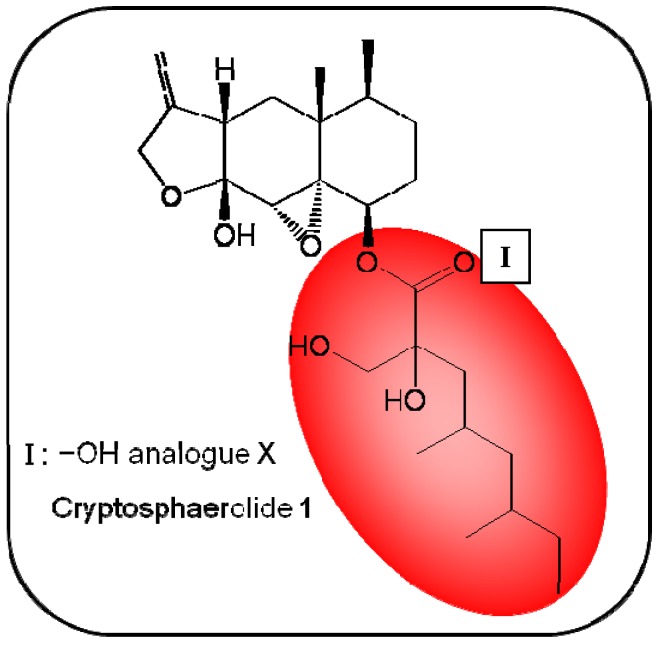
Summary of structure-activity relationship (SAR) results of the marine compound cyptosphaerolide (**1**) concerning the observed anti-cancer cytotoxicity. The pharmacophores identified during the studies are highlighted in color (**X**: inactive; 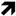, **=**, 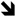: higher, similar, lower cytotoxicity).

Fenical *et al.* isolated cryptosphaerolide (**1**, Figure 1), an ester-substituted sesquiterpenoid, in 2010 from the ascomycete strain CNL-523 (*Cryptosphaeria* sp.) [26]. This marine product exerts cytotoxicity (IC_50_ of 4.5 μM) on the HCT-116 colon carcinoma cell line. A biochemical study revealed that this compound inhibited myeloid leukemia cell differentiation protein Mcl-1, a critical player involved in life/death decisions of individual cells [27], with an IC_50_ of 11.4 μM. Studies on a hydrolyzed analog of this compound demonstrated that the presence of a hydroxylated ester side chain, linked to the core sesquiterpenoid group, is responsible for the observed anti-cancer activity [26] (Figure 2).

### 2.2. Manzamine A *(**2**)*

The alkaloid manzamine A (**2**, Figure 1), which has been isolated from various marine sponges, exerted a cytotoxic effect against AsPC-1 pancreatic cancer cells, with an IC_50_ in a range of 4.2 μM, after 3 days of treatment [28]. Additionally, it was reported that manzamine A inhibited AsPC-1pancreatic cancer cell migration *in vitro*, and it decreased their overall metastatic potential. Fluorescent microscopy after staining with annexin V indicated an onset of apoptosis.

**Figure 3:**
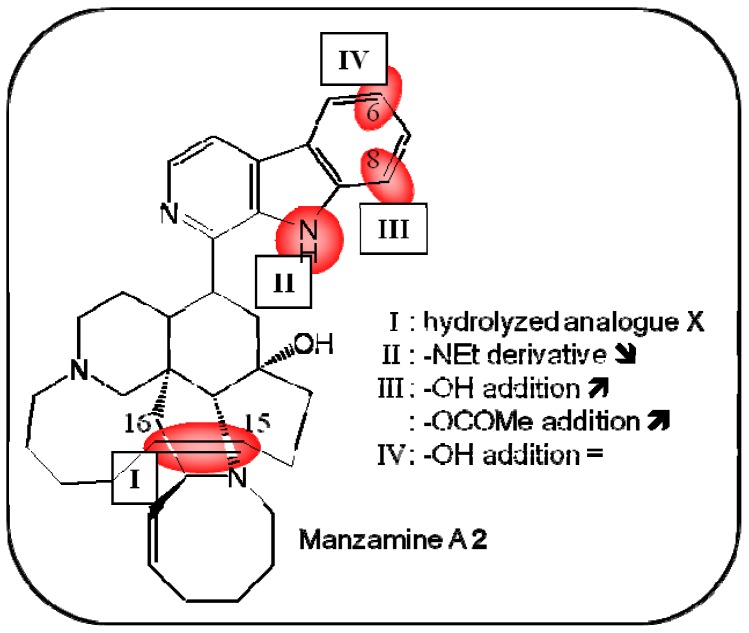
Summary of structure-activity relationship (SAR) results of the marine compound manzamine A (**2**) concerning the observed anti-cancer cytotoxicity. The pharmacophores identified during the studies are highlighted in color (**X**: inactive; 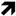, **=**, 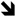: higher, similar, lower cytotoxicity).

Wright’s group reported the antimetastatic and proapoptotic effects of this alkaloid. Consequently, manzamine A can be used in combination of therapies, as it sensitizes cancer cells to TRAIL-induced apoptosis, an effect resulting from the inhibition of glycogen synthase kinase GSK3β [28]. Structure-activity-relationship studies revealed that neither the additional hydroxy group at position 8, nor a small variation in the amine group excessively altered the activity of the compound (Figure 3). Instead, the double bond between positions 15 and 16 was found to be crucial for the observed activity, as described by Hamann *et al.*, who indicated that the phenyl group of the heterocycle carboline group fits well into a pocket of this enzyme. Additionally, manzamine A inhibited cyclin-dependent kinase (CDK) 5, which decreased tau hyperphosphorylation in human neuroblastoma cells [29].

### 2.3. Cholestanes *(**3**)*

Polyoxygenated cholestanes **3** (Figure 1) were isolated from the sea whip *Leptogorgia sarmentosa*. Four of these steroids exhibited cytotoxic activity against mouse lymphoid neoplasma (P-388), human lung carcinoma (A 549), human colon carcinoma (HT-29) and human melanoma (MEL 28) cells, with an ED_50_ in the range of 1 μg/mL [30].

**Figure 4:**
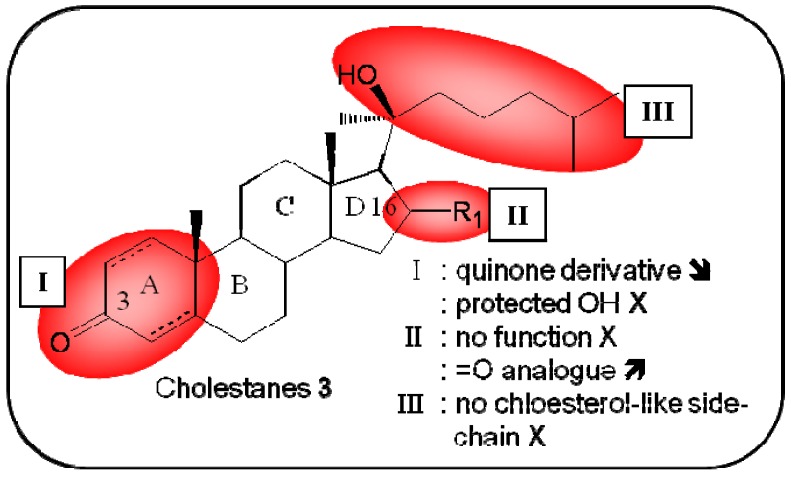
Summary of structure-activity relationship (SAR) results of the marine compounds cholestanes (**3**) concerning the observed anti-cancer cytotoxicity. The pharmacophores identified during the studies are highlighted in color (**X**: inactive; 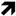, **=**, 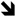: higher, similar, lower cytotoxicity).

Because this potent bioactive product can only be obtained in small quantities from marine sources, Kongkathip’s research group developed a method to produce these cholestanes synthetically. These steroids have an α,β-unsaturated ketone that exhibited significant cytotoxicity activity against human lung cancer cell lines (NCI) (IC_50_ of 6.2–10.5 μM), moderate activity against MCF7 breast cancer cell lines (IC_50_ of 30.7–31.4 μM) and human oral cancer KB (IC_50_ of 41.7–42.2 μM) cell lines [31]. Updated structure-activity-relationship studies have been recently published (Figure 4) and Kongkathip *et al.* reported that the hydroxyl groups at the C-3 and C-16 positions, as well as the cholesterol-like side chain, are crucial for the cytotoxic activity observed with these four steroids. Interestingly, a steroid with an aromatic A-ring exhibited the most potent cytotoxic effects, greater than that of the natural derivatives, against MCF7 and KB cell lines [32]. The biological pathway affected by this group of steroids remains unidentified.

### 2.4. epi-Malyngamide C *(**4**)*

**Figure 5:**
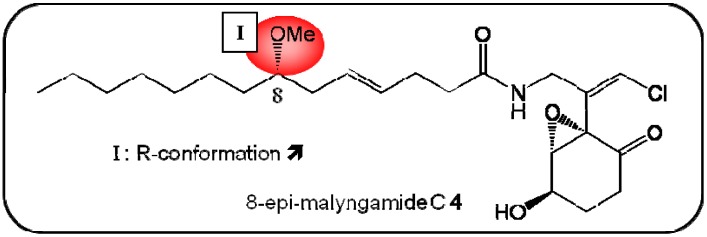
Summary of structure-activity relationship (SAR) results of the marine compound 8-epi-malyngamide C (**4**) concerning the observed anti-cancer cytotoxicity. The pharmacophores identified during the studies are highlighted in color (**X**: inactive; 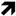, **=**, 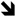: higher, similar, lower cytotoxicity).

Malyngamide C, a chlorinated amide derivative of lyngbic acid, was first isolated by Moor’s research team in 1985, from the cyanobacterium *Lynbya majuscula* [33]. In 2010, an epi-isomer of malyngamide C (**4**) was isolated and identified. Kwan *et al.* evaluated the bioactivity of this new stereoisomer and compared it to the activity of the native compound. The epi-isomer was less cytotoxic to HT-29 colon cancer cells [IC_50_ values of 5.2 and 15.4 (*epi*-isomer), respectively] [34]. The *R*-configuration of the alcohol group on the six-membered cyclic ketone ring is crucial for cytotoxic activity (Figure 5). To date, no studies on the signaling pathway used by malyngamides have been published.

### 2.5. HESA-A, a Drug from Herbal-Marine Origin

HESA-A (patented by researchers in Iran) is composed of both plant and marine materials, including material from *Penaeus latisculatus *(king prawn), *Carum carvi* (Persian cumin) and *Apiumgraveolens* (celery). HESA-A consists of both mineral and organic constituents and a small amount of water (45%, 50% and 5%, respectively) [35]. The exact biological targets of HESA-A have not been determined to date, but it is thought that this multi-component drug acts using a variety of pharmacological mechanisms [36]. Its efficiency as a non-toxic, chemotherapeutic agent has been confirmed recently in various *in vivo* and pre-clinical studies [35,37,38,39,40].

### 2.6. Spongistatin 1 *(**5**)*

The Pettit group isolated the macrocyclic lactone spongistatin 1 (**5**) from a marine sponge of the genus *Spongia* in 1993; this marine lactone exerted strong cytotoxicity on a panel of 60 types of human cancer cells [41]. Spongistatin 1 was reported to inhibit glutamate-induced tubulin polymerization (IC_50_ of 3.6 μM in PtK1 kangaroo rat kidney cells) through its interaction with the *Vinca* alkaloid domain of tubulin, which leads to the inhibition of mitosis [42].

After treatment of A549 lung cancer cells with spongistatin 1 (1 nM), cell cycle arrest at the G2-M phase, the simultaneous up-regulation of GADD45α-γ and down-regulation of c-Myc were observed [43]. Various studies have reported that this marine product triggers caspase-dependent apoptosis in leukemia cells, even in primary leukemia cell lines, at low concentrations (1 nM) [43,44,45].

**Figure 6:**
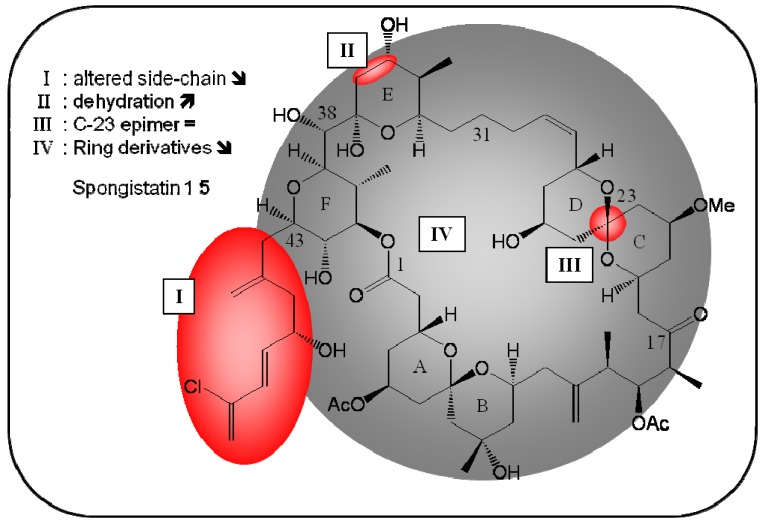
Summary of structure-activity relationship (SAR) results of the marine compound spongistatin 1 (**5**) concerning the observed anti-cancer cytotoxicity. The pharmacophores identified during the studies are highlighted in color (**X**: inactive; 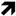, **=**, 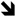: higher, similar, lower cytotoxicity).

This compound did not induce significant apoptosis in healthy peripheral blood cells, highlighting its potential use as a therapeutic drug [44]. Several structure-activity-relationship studies have been conducted to date (Figure 6); Kishi *et al.* noted that a C-23 epimer and spongistatin 1 had similar cytotoxic effects [46]. Paterson *et al.* reported that dehydration of the E-ring (C35-C36) led to an increase in cytotoxic potency, but that altering the side chain resulted in an important loss of activity [47]. A hydrogen-chlorine substitution in spongistatin 1 resulted in a 10-fold reduction in cytotoxicity [41]. In 2008, Heathcock *et al.* evaluated the toxicity of acyclic spongistatin 2 analogs, which contained only the E- and F-rings, as well as cyclic EF, ABEF and ABCD ring derivatives. In all derivatives tested, cytotoxicity was lost [48]. More recently, Smith *et al.* noted that an ABEF analog had cytotoxic effects when used in the nanomolar range, although its potency was 1,000 times weaker than that of spongistatin 1 [49]. In summary, these results demonstrate that the ABEF ring system, as well as the triene side chain, are crucial for spongistatin 1 cytotoxicity.

### 2.7. Bromopyrrole Akaloids

Marine sponges from the genera *Agelas*, *Axinella* and *Hymeniacidon* are known to synthesize bromopyrrole alkaloids [50,51]. In 1990, various compounds from a similar chemical class, namely hymenialdisine (**6**), debromo-hymeniaidisine (**7**) and agelasine G (**8**) were shown to exert significant cytotoxic activity against murine lymphoma cells (ED_50_ of 2.0–3.1 μg/mL). In contrast to these compounds, axinohydantoin (**9**) was significantly less active (IC_50_ of 18 μg/mL) [51,52]. Additionally, dibromophakellstatin (**10**) exhibited cytotoxicity against various human cancer cells at sub-micromolar concentrations, but replacement of the urea group with a guanidine resulted in a decrease in activity [53]. In 2010, Xu *et al. *demonstrated that bromopyrrole alkaloids have anti-cancer activity *in vivo*. This was observed with a novel bromopyrrole, *N*-(4,5-dibromopyrrole-2-carbonyl)-L-aminoisovaleric acid methyl ester (**11**). This compound inhibited the proliferation of human cancer cells *in vitro* (IC_50_ of 3.8–17.2 μg/mL) and in xenografted mice (MIC at 40 mg/kg). It has also been reported that bromopyrrole triggered cell cycle arrest in the G1 phase, and that it induced caspase-dependent apoptosis [54]. Another compound, sceptrin (**12**), composed of two bromopyrroles bound to a cyclobutane unit, halted cell motility in a variety of cancer cells (at 40 μM) [55] but had no effect on cell proliferation or survival [55]. It has been reported to be noncytotoxic to monkey kidney cells (at 200 μg/disk) [56]. The three sceptrin derivatives, namely nakamuric acid (**13**), its methyl ester derivative and debromosceptrin, exhibited lower anti-motility effects compared to sceptrin [55]. However, detailed mechanistic studies on the pathways affected by sceptrin still need to be conducted.

### 2.8. β-Carbolines

Crews *et al.* isolated a novel β-carboline, named hyrtiocarboline (**14**), from the marine sponge *Hyrtios reticulates*. This β-carboline exhibited antiproliferative activity when applied to a panel of 13 cancer cell types, with an IC_50_ as low as 1.2 μg/mL for non-small cell lung cancer (H522-T1) cells [57]. The core structure of β-carboline may represent a promising chemical class, as reported in several publications [58,59]. To date, a few β-carboline alkaloids have been isolated from marine sources, namely 5-bromo-8-methoxy-1-methyl-β-carboline (**15**) [60], norharman (**16**) [61], 2-methyl-eudistomins-J-D (**17**–**18**), and 14-methyleudistomin C (**19**) [62]. Only the latter had cytotoxic effects in sub-micromolar range. Further investigations on the affected pathways need to be conducted.

### 2.9. Makaluvamine Analogs

**Figure 7:**
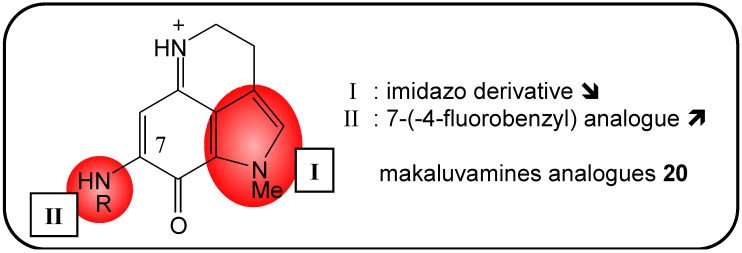
Summary of structure-activity relationship (SAR) results of the marine compounds makaluvamines (**20**) concerning the observed anti-cancer cytotoxicity. The pharmacophores identified during the studies are highlighted in color (**X**: inactive; 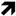, **=**, 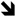: higher, similar, lower cytotoxicity).

Makaluvamines (**20**) (a type of pyrroloiminoquinone) were first isolated from marine sponges belonging to the genera *Zyzzya* and *Histodermella* [63,64]. These products exert cytotoxic activity against various cell lines by inhibiting DNA topoisomerase II [65]. Anti-cancer activity was observed with bioactive synthetic analogs of these compounds. Imidazoquinoxalinone derivatives **21** have been reported to be less active due to the presence of an electro-deficient benzimidazole ring; the naturally occurring compound contains an indole ring. Under physiological conditions, the cationic makaluvamines were reported to be active, whereas imidazoquinoxalinones analogs are not charged, which explains the decreased activity of the latter [66,67]. Velu *et al. *evaluated the biological activity of makaluvamines containing various substitutions at the 7-position of the pyrroloiminoquinone ring. Observations from many natural derivatives allowed them to conclude that the presence of functional groups at this position greatly increased cytotoxic potential (Figure 7). *In vitro* testing with a NCI panel of 60 human cancer cell types indicated that the 7-benzyl- and 7-(4-fluorobenzyl) analogs (BA-TPQ and FBA-TPQ, respectively) exhibited the greatest cytotoxic effects [68,69]. These promising results lead various groups to perform pre-clinical studies. In breast cancer cell lines, both products significantly decreased cancer cell growth, induced apoptosis and caused cell cycle arrest at submicromolar concentrations (0.5 μM). Additionally, it has been reported that the anticancer activity was independent from the activity of p53 in cancer cells [70,71]. FBA-TPQ also strongly inhibited cancer cell proliferation, activated apoptosis and caused cell cycle arrest in prostate cancer cells, in the low micromolar range (2 μM). Furthermore, androgen receptor (AR) and prostate-specific antigen (PSA) levels, as well as the expression of apoptosis-related proteins were reduced [72]. A pharmacological study conducted in mice revealed that intravenously-injected BA-TPQ accumulated in the lungs, kidneys and spleen; it even reached low concentrations in the brain. However, this product was systemically toxic (indicated by animal weight loss) when administered at a concentration of 10 mg/kg [73]. The data presented here clearly indicated that makaluvamine analogs represent a promising choice for future clinical trails, and they may promote the development of novel anticancer drugs.

### 2.10. Cyclopentenones

In 1977, cyclopentenones were shown to possess antitumor properties and to down-regulate cellular metabolism [74,75]. It is has been noted that prostaglandins A_1_, A_2_ and J_2_ (compounds **22**–**24**), which are known cancer-proliferation inhibitors, carry an a,b-unsaturated cyclopentenone ring linked to alkyl chains [76]. It is not surprising that the bioactivity of prostaglandins is mainly due to the presence of the cyclopentenone ring. An α,β-unsaturated ketone group is thought to act as an important alkylating center through a Michael-type reaction with cysteine [74,75,76]. Detailed mechanistic studies indicated that cyclopentenone (**25**) caused cell cycle arrest by the repression of cyclin D1, inhibited constitutive NF-kB activity and lead to the induction of apoptosis [77,78]. Novel cytotoxic cyclopentenones, namely didemnenone (**26**) and trichoderone (**27**), have been isolated from the didemnid ascidian *Lissoclinum* sp., and from the marine-derived fungus *Trichoderma* sp. [79,80]. In both cases, the IC_50_ was in the micromolar range. These findings highlight the anticancer potential of cyclopentenone groups, making them interesting compounds on which to focus future studies. 

### 2.11. Heteronemin and Semi-Synthetic Derivatives

The pentacyclic scalarane heteronemin (**28**) was first isolated in large quantities from the sponges *Heteronema erecta* and *Hytios* sp. in 1976 [81]. Crews *et al.* reported heteronemin had cytotoxic effects when applied to brine shrimp and giant kelp (*Macrocystis pyrifera)* gametes [82]. In cytotoxicity assays, this sesterterpene induced cell death in human thyroid carcinoma cells and an analog, 12-deacetoxy-21-hydroxyheteronemin, exhibited significant cytotoxicity against K562 cells [83,84]. The biological pathways affected by this marine product have since been identified. It has also been shown that heteronemin exerts antitubercular activity by inhibiting farnesyl transferase [85,86]. Furthermore, results from our laboratory have clearly shown that heteronemin attenuates NF-κB pathway activation through the down-regulation of proteasome activity [87]. Heteronemin triggered caspase-dependent apoptosis in K562 cells, and it sensitized K562 cells to TNFα-induced apoptosis [87].

**Figure 8:**
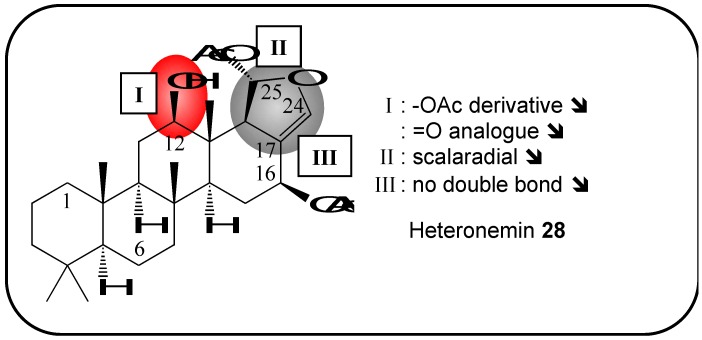
Summary of structure-activity relationship (SAR) results of the marine compound heteronemin (**28**) concerning the observed anti-cancer cytotoxicity. The pharmacophores identified during the studies are highlighted in color (**X**: inactive; 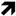, **=**, 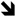: higher, similar, lower cytotoxicity).

Two structure-activity-relationship studies were published in 2009, with focus on the anti-carcinogenic effects induced by this compound [88,89] (Figure 8). Despite the fact that half of the tested analogs showed little or no cytotoxicity towards normal human oral fibroblasts or monkey kidney epithelial cells, these studies demonstrated that the oxygen atoms at positions C-25 and C-16 were crucial for the cytotoxic activity of heteronemin, whereas the double bond at position C-17-C-24 was of marginal importance [88,89]. To summarize, these promising results indicate that heteronemin and some of its derivatives represent interesting candidates for future chemotherapeutic drug research.

### 2.12. Latrunculin A and B

Latrunculins A and B (compounds **29**,**30**) were first isolated from the Red Sea sponge *Negombata magnifica *[90,91]. The core structure of both compounds consists of a macrolide fused to a tetrahydropyran moiety, where the latter is linked to a 2-thiazolidinone side chain. These were the first marine natural products reported to bind reversibly to actin, leading to its disorganization [91]. These compounds also exerted potent activity on the angiogenesis, migration and proliferation of cells [92,93]. Latrunculin A has a therapeutic index (T/C) of 146% in mice [94], a remarkable result, considering the actin-active agents jaspamide and cucurbitacin did not have comparable therapeutic indices in case study conducted by scientists in the Developmental Therapeutics Program (DTP) at the NCI [95,96]. X-ray crystallography demonstrated that 2-thiazolidinone fits perfectly into actin pockets, and each polar oxygen, except for the O_2_-ester, forms a hydrogen bond with actin [97].

**Figure 9:**
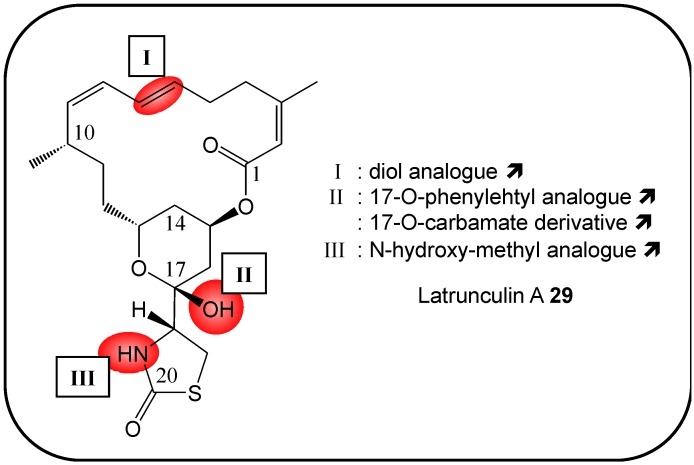
Summary of structure-activity relationship (SAR) results of the marine compound latrunculin A (**29**) concerning the observed anti-cancer cytotoxicity. The pharmacophores identified during the studies are highlighted in color (**X**: inactive; 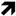, **=**, 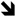: higher, similar, lower cytotoxicity).

Several structure-activity-relationship studies have been completed [92,98,99,100,101]. The deletion of methyl groups from the macrocycle group of latrunculin B resulted in an increased activity and a simplified synthetic target [99]. The composition of latrunculin A’s 16-membered macrolide, as well as the conformation of its thiazolidinone ring play critical roles in its anticancer activities, as observed with human solid cancer cell lines HCT-116 and MDA-MB-435 [98]. Carbamate derivatives exerted 2.5- to 5-fold greater anti-invasive activity against the extremely metastatic human prostate PC-3M cancer cells, with lower actin binding properties [101] (Figure 9). El Sayed *et al.* demonstrated that both 17-O-phenylethyl- and *N*-hydroxymethyl-analogs of latrunculin A had higher activity than the parent product [100].

### 2.13. Dermacozines

**Figure 10:**
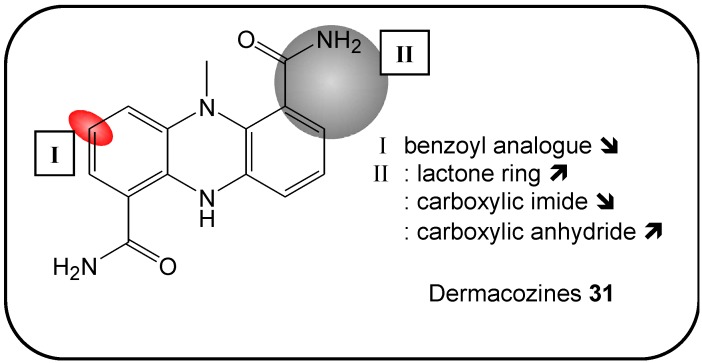
Summary of structure-activity relationship (SAR) results of the marine compounds dermacozines (**31**) concerning the observed anti-cancer cytotoxicity. The pharmacophores identified during the studies are highlighted in color (**X**: inactive; 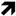, **=**, 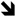: higher, similar, lower cytotoxicity).

Dermacozines (**31**), phenazine-type pigments, have been isolated from marine actinomycetes isolated from Mariana Trench sediment from a depth of 10.898 meters by Jaspar’s research group [102]. This novel class of phenazines has been characterized and confirmed through in-depth analysis of 1D-,2D-NMR data combined to high-resolution MS, UV-data and CD spectroscopy. Hence these marine products exerted a cytotoxicity activity *versus* leukemic K562 cancer cells with an IC_50_-range from 7 to 220 mM. A structure-activity-relationship study showed that a carboxamide moiety nor a lactone ring or a benzyl function majorly affected the observed cytotoxicity of the products (Figure 10). However, an additional carboxylic anhydride linked to the phenazine core structure led to a 20-fold increase in observed cytotoxicity in contrast to an imide ring. The latter did not alter the observed activity [102]. Further mechanistic studies on the pathways affected by dermacozines need to be conducted.

## 4. Conclusions

This review provides insight into the current literature regarding marine natural products and their derivatives which was published in 2010. The data presented here indicate the great value of natural marine products, as well as their synthetic analogs. The data suggest that these synthetic analogs, in particular, could be important candidates for further studies involving structural modifications to improve the pharmacological profile of native marine metabolites. Furthermore, a simplified analog with equipotent activity can lead to the development of a simplistic synthesis process, which would guarantee a sufficient supply if bioactive products for further investigation. In conclusion, the isolation or modification of novel marine products, as well as their analogs, and the subsequent evaluation of their bioactivity will push the discovery of novel promising chemotherapeutic drugs forward.
